# Genome-wide association study for *Streptococcus iniae* in Nile tilapia (*Oreochromis niloticus*) identifies a significant QTL for disease resistance

**DOI:** 10.3389/fgene.2023.1078381

**Published:** 2023-03-02

**Authors:** Sergio Vela-Avitúa, Benjamin R. LaFrentz, Carlos A. Lozano, Craig A. Shoemaker, Jose Fernando Ospina-Arango, Benjamin H. Beck, Morten Rye

**Affiliations:** ^1^ Benchmark Genetics Norway AS, Bergen, Norway; ^2^ United States Department of Agriculture-Agricultural Research Service (USDA-ARS), Aquatic Animal Health Research Unit, Auburn, AL, United States; ^3^ Spring Genetics, Miami, FL, United States

**Keywords:** genome-wide association study (GWAS), *Streptococcus iniae*, Nile tilapia, survival, quantitative trait loci (QTL), marker assisted selection (MAS)

## Abstract

*Streptococcus iniae* is a problematic gram-positive bacterium negatively affecting Nile tilapia (*Oreochromis niloticus*), one of the main aquacultural species produced worldwide. The aim of this study was to identify the genetic architecture of survival to *S. iniae* and identify single nucleotide polymorphism (SNPs) linked to quantitative trait loci (QTL) related to survival to *S. iniae* challenge. With this purpose, Nile tilapia from the Spring Genetics breeding program were sent to a controlled *S. iniae* challenge test where phenotypes were scored as dead for fish that died during challenge test and survivors for the fish alive at the termination of the test. Additionally, fin-clip samples from all fish in the test were collected for DNA extraction. Out of 1904 fish in the challenge test, tissue samples of 321 fish were sent for genotyping using double digest restriction site associated DNA sequencing (ddRADseq). After quality control and filtering, 9,085 SNPs were used to perform a genome-wide association study (GWAS). A significant signal in LG8 was observed indicating association with survival to *S. iniae* challenge, with SNPs explaining from 12% to 26% of the genetic variance. To demonstrate the usefulness of marker assisted selection (MAS) to selectively breed fish for survival to *S. iniae,* offspring of breeding candidates classified as “resistant” and “susceptible” based on haplotypes of the four most significant markers were sent to a controlled *S. iniae* challenge test. At the end of the test, the differences in mortality between the two groups were strikingly different with a final cumulative percent mortality of less than 1% and 73% for offspring from “resistant” and “susceptible” parents, respectively. These results demonstrate that MAS for improved resistance to *S. iniae* is feasible.

## 1 Introduction

Due to its high adaptability, Nile Tilapia (*Oreochromis niloticus*) is among the three most important cultured aquatic species with more than four and a half million tons produced worldwide in 2020 (FAO, 2022). Tilapia farming has grown at a faster rate than overall aquaculture in the past two decades, leading to more intensive production but also to an increase in occurrence of diseases (Xu and Ming, 2018; Miao and Wang, 2020). Streptococcal disease, caused by *Streptococcus iniae, S. agalactiae* and *S. dysgalactiae*, is a major bacterial disease affecting tilapia populations worldwide. Due high morbidity and mortality rates, this disease causes a substantial negative economic impact (estimated at US$ 1 billion) to the global tilapia industry (Shoemaker et al., 2017b). In 1997, the annual impact of *S. iniae* in the United States was estimated at US$ 10 million (Shoemaker et al., 2001). Selective breeding for disease resistance is a possible solution to overcome problems caused by bacterial diseases in tilapia, particularly since large additive genetic variation was recently found for *S. iniae* (LaFrentz et al., 2016; Shoemaker et al., 2017a) and selective breeding has shown to increase resistance in Nile tilapia (*O. niloticus*) populations (LaFrentz et al., 2020). In Nile tilapia, selective breeding programs has primarily been based on pedigree (Ponzoni et al., 2010; Thodesen Da-Yong Ma et al., 2011; Trong et al., 2013; Bentsen et al., 2017; Shoemaker et al., 2022). However, the development of new technologies facilitating marker assisted selection (MAS) and genomic selection (GS) may increase genetic gain through improved prediction accuracy and by allowing to capitalise on all the genetic variation when breeding candidates have not been recorded for the traits under selection (Houston, 2017). MAS can be used to improve disease resistance if quantitative trait loci (QTL) are found for the specific disease targeted and they explain a large amount of the genetic variation. In Atlantic salmon, a major QTL that explained 80%–98% of the genetic variation for infectious pancreatic necrosis (IPN) resistance was found and is currently widely used for disease control (Houston et al., 2008; Moen et al., 2009; Moen et al., 2015). QTL that affects disease resistance have been found for some fish species (Fuji et al., 2006; Gilbey et al., 2006; Moen et al., 2007; Vallejo et al., 2014; Gonen et al., 2015), but the use of MAS based on single QTL is not always successful in animal breeding due to the polygenic genetic architecture of most economically important traits (Meuwissen et al., 2013). To date, no major QTL for resistance to bacterial diseases has been reported in Nile tilapia. In a genome-wide association study (GWAS) for *S. agalactiae* capsular type 1a, Lu et al. (2020) identified seven trait-related SNPs on four different chromosomes that together explained 1% of the genetic variation and concluded that resistance to *S. agalactiae* 1a is polygenic. The objective of this study was to investigate the genetic architecture of and possibly identify molecular markers linked to resistance to *S. iniae* in Nile tilapia.

## 2 Materials and methods

### 2.1 Family production, challenge testing and training data selection

Fish from the fourth generation (G4) of the spring Genetics Nile tilapia breeding program were used for the GWAS. A total of 144 full sib families were produced by natural mating in single pair breeding units using a nested mating design (144 dam with 72 sires) and reared separately until tagging as previously described (LaFrentz et al., 2016; Shoemaker et al., 2017a; LaFrentz et al., 2020). At tagging, tissue samples from the pelvic fin of all fish destined to participate in the challenge tests were taken and stored in 97% ethanol in separate 1.5 mL Eppendorf tubes with individual identification and kept at −20°C. After tagging, representatives of all families were stocked at Spring Genetics’ Nile tilapia breeding program facilities in Homestead, Florida (FL) and reared as breeding candidates. Siblings of G4 breeding candidates representing all families were transported to the USDA-ARS Aquatic Animal Health Research Unit (AAHRU) in Auburn, Alabama and subjected to two separate controlled *Streptococcus* spp. challenge tests (*S. iniae* and *S. agalactiae* 1b). Results and details of these two challenge tests have been previously published by Shoemaker et al. (2017a). For *S. iniae*, fish from all families were challenged intraperitoneally with diluted *S. iniae* culture (see details from section 2.5 below).

After finalization of the challenge test a training population was constructed with a subsample of the fish that participated in the *S. iniae* challenge test. Families were grouped according to their genetic distances estimated from pedigree. From resulting groups, half-sib families that maximized phenotypic variation were selected and within each family individuals were randomly sampled. After this process, 321 individuals representing 39 families were selected to create the training population for *S. iniae* (Table 1). Survival recorded as alive at end of challenge test (scored 1) or dead during trial (scored 0) was used as the phenotype for statistical analysis.

**TABLE 1:** Total number of families, average number of individuals per family, and total number of individuals phenotyped and genotyped of the first challenge test of *S. iniae* used for the GWAS.

	Phenotyped	Genotyped
Number of families	144	39
Total number of fish	1904	321
Mean number of fish/family	18.6	8.5
Mean survival (sd)	0.54 (0.5)	0.52 (0.5)

### 2.2 Genotyping and variant calling

Tissue samples of 321 Nile tilapia challenged with *S. iniae* were genotyped together with 777 Nile tilapia from the same population using ddRADseq. Tissue samples were sent to LGC Genomics GmbH (Germany) for DNA extraction, library preparation and sequencing. In brief, DNA was extracted from fish fin clips (16–25 mm^2^) using sbeadex livestock kit (LGC Genomics GmbH, Germany). 100–200 ng of genomic DNA were digested with MsII (NEB) in NEB4 buffer. After inactivation of restriction enzyme, 10 µL of each restriction digest were transferred to a new 96 well PCR plate with one of 96 inline barcoded forward blunt adaptors, and ligation master mix. After ligation, all reactions were purified using Agencourt AMPure XP beads (Beckman Coulter, United States of America) and libraries eluted in Tris buffer. Ten µl of each of the 96 Libraries were separately amplified in 20 µL PCR reactions using MyTaq (Bioline GmbH, Germany) and standard Illumina TrueSeq amplification primers (Illumina Inc., United States) limiting the cycle number to 14 cycles. Five µl from each of the 96 amplified libraries were pooled, PCR primer and small amplicons were removed using Agencourt AMPure XP beads (Beckman Coulter, United States) and PCR enzyme removed by an additional purification on Qiagen MinElute Columns (Qiagen, United States). Pooled libraries were eluted in a final volume of 20 µL Tris Buffer (5 mM Tris/HCl pH:9). Normalisation was done using Evrogen Trimmer Kit (Evrogen, Russia). Normalized library pools were re-amplified in 100 µL PCR reactions using MyTaq (Bioline GmbH, Germany) and limiting cycle number to 14 cycles. For each pool a different i5-adaptor primer was used to include i5-indices into the libraries. Libraries were size selected on Blue Pippin and LMP-agarose gel, keeping fragments between two hundred and four hundred base pairs.

Sequencing was done on an Illumina NextSeq 500 V2 platform (Illumina Inc., United States), resulting in ∼1.5 M 150 bp single-ended reads per sample. Libraries were demultiplexed using Illumina’s bcl2fastq 2.17.1.14 software^1^ and reads processed with custom Python scripts to sort them into samples removing barcode sequences. Adapter sequences were removed with cutadapt (Martin, 2011) discarding reads shorter than 20 bp. All reads were filtered for restriction enzyme cut sites. The remaining reads were quality-trimmed by LGC proprietary software, removing all reads with an average Phred score below 20 and removing low quality tails where the average Phred score fell below 20 over a window of ten bases, as well as discarding reads containing more than 1 undetermined base (N).

The pre-processed reads for all samples were aligned against the *Oreochromis niloticus* reference genome from NCBI accession number GCA_001858045.1 ASM185804v2 (Conte et al., 2017) using BWA-MEM version 0.7.5a (Li and Durbin, 2010). All resulting BAM files were sorted and merged using Samtools (Danecek et al., 2021) and Picard^2^. Variant calling and genotyping was performed with Freebayes v1.0.2-16 (Garrison and Marth, 2012) filtered for minimum coverage (10 reads), minimum allele frequency (>2%) and minimum sample coverage (at least 600 samples). After processing, ∼83 K SNPs (83,752 SNPs) were identified for all 1,098 Nile tilapia samples.

### 2.3 SNP filtering

After allele calling, the 321 Nile tilapia samples from *S. iniae* challenge test were filtered using Plink v1.9 (Chang et al., 2015). SNPs were filtered for minimum allele frequency (MAF) larger than 0.05, allele call larger than 0.1 and Hardy Weinberg equilibrium p < 1e-6 resulting in 9,085 SNPs after filtering. Samples were filtered for genotype rate (>90%) and heterozygosity (<±3sd of population), after filtering no samples were removed.

### 2.4 Statistical analysis

GWAS was performed using the linear mixed animal model implemented in GCTA software (Yang et al., 2011) with the option of leaving one chromosome out (MLMA-LOCO). In MLMA-LOCO polygenic effect is estimated as accumulated effect of markers across all linkage groups (LG) except the LG where the candidate marker is located. The general model fitted was:

y=μ+Xb+Za+e (1)

Where ***y*** is the vector of phenotypic records (i.e., survival score 0 or 1), ***μ*** was the overall mean, ***b*** was the vector of unknown additive effect of the evaluated SNP, ***a*** was the vector of additive polygenic effects with distribution $∼N\left(0,G\sigma_{a}^{2}\right)$ where G is the genomic relationship matrix as $G=\frac{WW^{\prime }}{2\sum p_{i}\left(1-p_{i}\right)}$ ; where W is standardized genotype and $p_{i}$ the frequency of the second allele at locus i (VanRaden, 2008) and ***e*** is a vector of residual effects $∼N\left(0,I\sigma_{e}^{2}\right)$ . ***X*** and ***Z*** are design matrices to the respective vectors ***b*** and ***a***. Statistical significance threshold for *p*-values was estimated using Bonferroni correction method for multiple testing with P≤0.01 . Genetic variance explained by a SNP was estimated as $\sigma_{SNP}^{2}=2pqa^{2}$ where *p* and *q* are allele frequencies and *a* is the estimated allele substitution effect.

### 2.5 Assortative mating using haplotypes and *S. iniae* challenge test

From 2019 onwards, Spring Genetics has implemented genomic selection for resistance to *S. agalactiae* and from 2020 to *Francisella oreintalis*; thus, male breeding candidates are routinely genotyped using a custom BMK Genetics 50K SNP array developed in collaboration with Neogen (Neogen Corp., Lexington, KY) which includes the ten most significant markers for survival to *S. iniae*. Additionally, Spring Genetics in collaboration with Neogen, designed a panel with 222 SNPs for parental assignment and traceability including the ten most significant markers for survival to *S. iniae* and is routinely used to perform MAS on females to *S. iniae*. Thus, on generation seven (G7), genotypes of breeding candidates from the Spring Genetics population were available. In total 768 candidate males were genotyped with the 50K SNP array, and 800 candidate females were genotyped with the parental assignment panel. Five months after the production of generation eight (G8) of the Spring Genetics’ breeding nucleus, experimental groups were produced to probe the effectiveness of MAS for selection to survival to *S. iniae*. For this purpose, using available genotypes of breeders that remained alive, fish were classified into “resistant” and “susceptible” groups, based on haplotypes of the four most significant markers. Individual pairs of breeders were subsequently stocked in separate hapas according to their group. Five “resistant” hapas and five “susceptible” hapas were stocked, allowing single pair mating. Due to low percentage of spawning caused by cold water temperatures during this period, fry from one “resistant” female and two “susceptible” females were collected (Table 2). Offspring from “resistant” and “susceptible” matings were reared in separate tanks until tagging. A total of 298 fish from the “resistant” group and 304 fish from the “susceptible” group were PIT tagged, tissue samples were taken for DNA (as described in 2.1) and individual weight and group were recorded. The average weight at tagging was 14.2 g for the “susceptible” group and 14.6 g for the “resistant” group. Fish from both groups were pooled and transported by truck from Spring Genetics to the USDA-ARS AAHRU to be challenged with *S. iniae*. Upon arrival fish were acclimated in a common 5,500 L tank for 28 days that was supplied with de-chlorinated municipal water with average temperature and dissolved oxygen of 26.3°C ± 1.1°C and 7.2 ± 0.5 ppm, respectively. Pilot challenges were conducted with sibs from both groups previously sent to the AAHRU to determine the dose of *S. iniae* to be used in trial. An archived stock of *S. iniae* generated following passage through tilapia was cultured as previously described by LaFrentz et al. (2016); Shoemaker et al. (2017a). A total of 590 fish (average weight of ∼40 g) were challenged with *S. iniae via* intraperitoneal injection with a dose of 5 × 10^6^ cfu fish^-1^ and all fish were placed into a single 5500 L tank. The temperature and dissolved oxygen averaged 27.7°C ± 0.1°C and 6.4 ± 0.4 ppm, respectively, during the challenge test. Following the challenge, moribund and dead fish were removed at least twice daily, with PIT tag numbers and date of removal recorded for each fish. Brain tissue samples were collected from 20% of the daily mortalities and plated onto sheep blood agar to confirm the cause of morbidity. At the end of the challenge test, all surviving fish were humanely euthanized and PIT tag numbers were recorded. Fish that lost PIT tags during the trial were excluded from analysis. Time to death of the two groups were compared using the Kaplan-Meier estimator (censor status = 1 for dead fish, censor status = 0, for fish alive). The survival during trial (alive = 1 or dead = 0) was used as the phenotype. All challenge procedures utilizing fish were approved by the USDA-ARS, AAHRU Institutional Animal Care and Use Committee.

**TABLE 2:** Breeders selected and stocked in breeding hapas for the QTL validation experiment.

Animal ID	Sex	Group	Number of *S. iniae* QTL markers		
			pp	qq	pq
G7B1-FemR1	Female	Resistant	3	0	1
G7B1-MalR1	Male	Resistant	4	0	0
G7B1-FemS1	Female	Susceptible	0	4	0
G7B1-FemS2	Female	Susceptible	0	4	0
G7B1-MalS1	Male	Susceptible	0	3	1

With pp, number of markers homozygous resistant; qq, number of markers homozygous susceptible; pq, number of markers heterozygous; Group, is the group into which fish were classified based on four selected markers (Table 3).

Tissue samples from all challenged fish were sent for genotyping using the custom SNP panel for parental assignment; offspring were assigned to their parents using hsphase package (Ferdosi et al., 2014) in R (R Core Team, 2013).

## 3 Results

### 3.1 Challenge test G4

Results of the challenge test for G4 were previously published (Shoemaker et al., 2017a). A brief summary is provided here for the reader. Most of the fish died on the first and second day after injection with *S. iniae*, and after day 5 daily mortalities were below 1%. Cumulative mortality reached 46% after 21 days. Brain tissue samples were collected from 265 fish that died during the trial and 99% of these samples yielded pure cultures of *S. iniae* confirming the cause of death. Heritability reported was high (h^2^ = 0.52 ± 0.12) (Shoemaker et al., 2017a).

### 3.2 Genome wide association study

**FIGURE 1:**
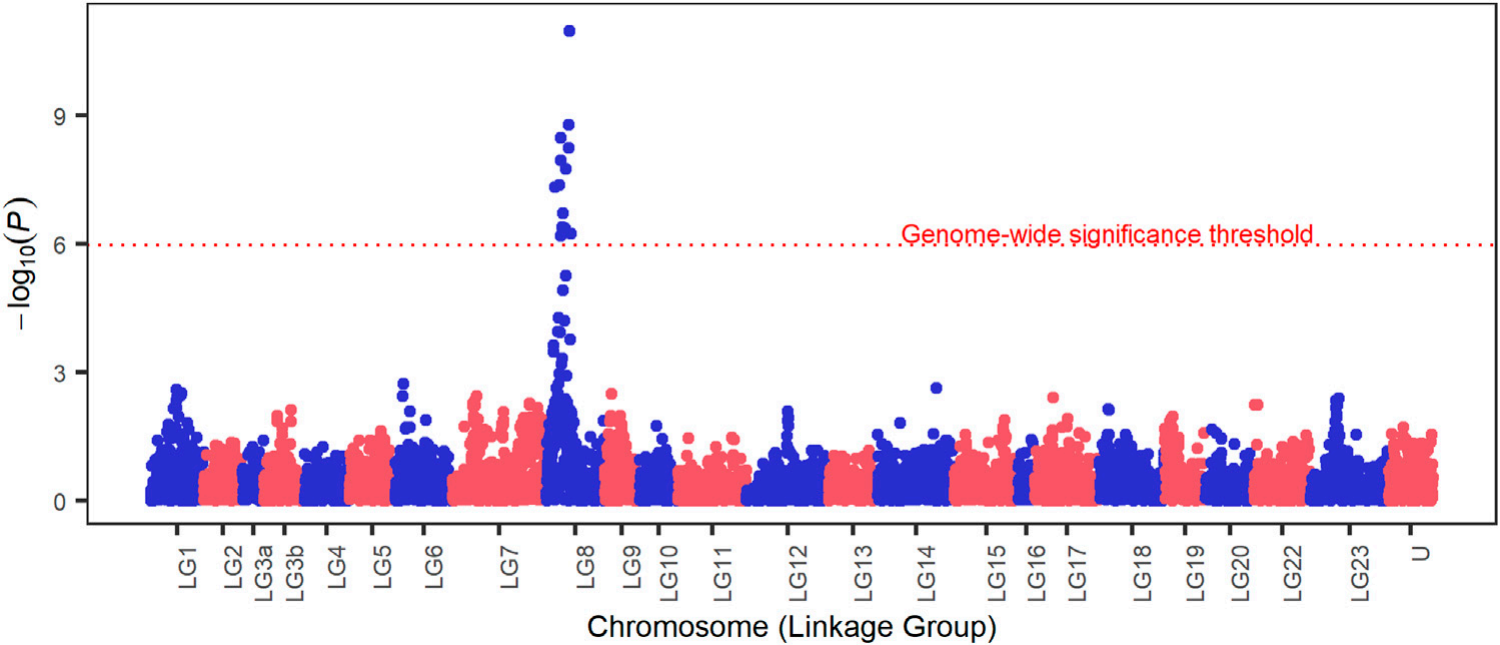
Manhattan plot for survival to *S. iniae* in Nile tilapia. On the y axis is the - *log_10_(p-values)* and the x axis is the chromosome (linkage group) position on the *Oreochromis niloticus* genome, U represents SNPs with unknown chromosome location. The dotted red line marks the genome-wide Bonferroni corrected threshold.

**FIGURE 2:**
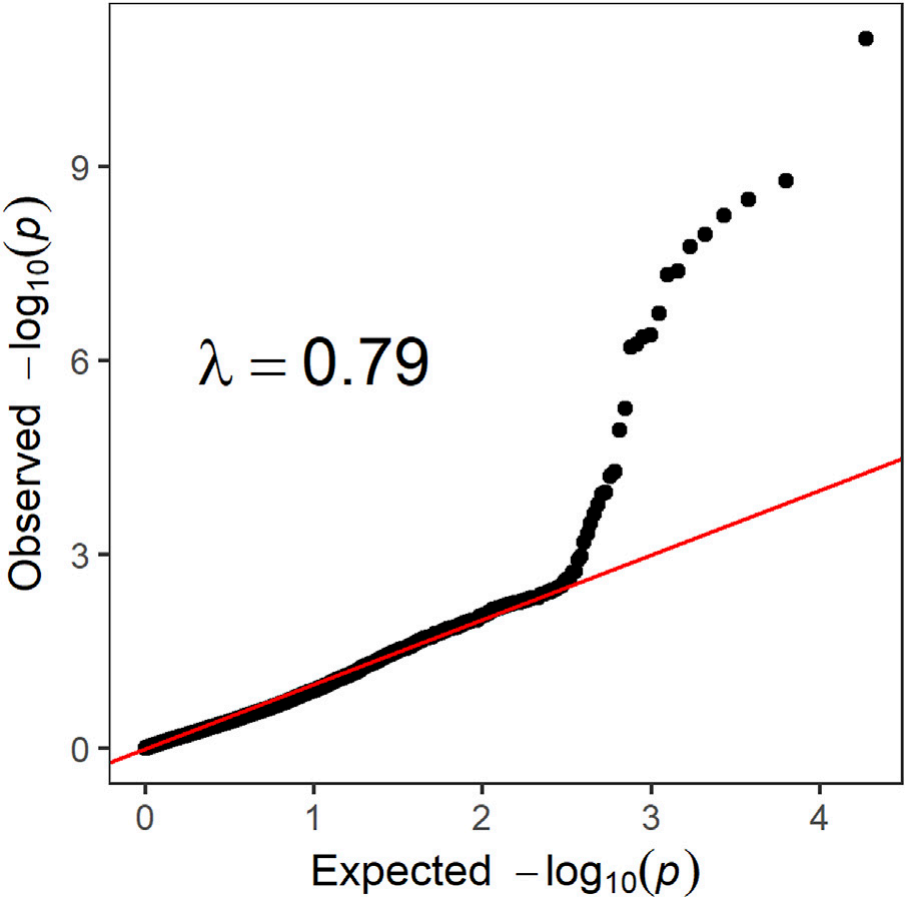
Quantile-quantile plot with the distribution of observed vs. expected -*log_10_(p)*.

The results of the GWAS analysis demonstrated a strong signal on LG8 indicating association with survival to *S. iniae* challenge test (Figure 1). Fourteen SNP markers overpassed the genome-wide threshold in a region comprising approximately 3.98 Mb on LG8, from position 5,545,222 to position 9,515,899. Details of significant markers are provided in Table 3, and these explained from 12% to 26% of the genetic variance. Genomic inflation factor λ=0.79 (Figure 2) indicated low population structure on the phenotypic data. To test the possibility of multiple QTLs in the significant region on LG8, the most significant SNP was included in the analysis as covariate, as result, none of the surrounding SNPs showed association with the trait (Figure 2).

**TABLE 3:** List of significant markers ordered according to their significance.

SNP ID	Chr	Position (bp)	A1	A2	MAF	α (±se)	p	%Var_P_	%Var_G_
NC_031973.1_9485417^*^	LG8	9485417	T	C	0.316	-0.298 (0.044)	1.00E-11	12.97%	25.66%
NC_031973.1_9167743^*†^	LG8	9167743	T	C	0.153	0.336 (0.056)	1.58E-09	12.88%	19.58%
NC_031973.1_7142946^*†^	LG8	7142946	T	C	0.298	-0.244 (0.041)	3.17E-09	8.71%	16.59%
NC_031973.1_9209387^*†^	LG8	9209387	G	A	0.150	0.337 (0.058)	5.45E-09	10.21%	19.25%
NC_031973.1_7142916^†^	LG8	7142916	G	A	0.171	-0.286 (0.050)	1.07E-08	8.59%	15.47%
NC_031973.1_7782524	LG8	7782524	C	T	0.147	0.325 (0.058)	1.68E-08	8.11%	17.69%
NC_031973.1_6323968	LG8	6323968	A	G	0.156	0.314 (0.057)	3.98E-08	8.15%	17.26%
NC_031973.1_5545222	LG8	5545222	G	A	0.146	-0.289 (0.053)	4.52E-08	8.27%	13.86%
NC_031973.1_5929441	LG8	5929441	G	A	0.153	0.298 (0.057)	1.80E-07	7.21%	15.31%
NC_031973.1_7497722	LG8	7497722	C	T	0.144	-0.274 (0.053)	1.84E-07	7.72%	12.29%
NC_031973.1_5908105	LG8	5908105	C	A	0.143	-0.285 (0.055)	2.73E-07	6.75%	13.25%
NC_031973.1_7357441	LG8	7357441	G	A	0.139	-0.270 (0.053)	3.89E-07	6.99%	11.56%
NC_031973.1_6392144	LG8	6392144	C	G	0.143	-0.280 (0.055)	3.97E-07	6.46%	12.80%
NC_031973.1_7775443	LG8	7775443	A	T	0.148	-0.267 (0.053)	4.23E-07	6.86%	11.95%

SNPs have been sorted according to their significance level, with Chr, chromosome (Linkage group) on the *Oreochromis niloticus* genome; A1, reference allele (minor); A2, second allele (major); MAF, minor allele frequency; α, allele substitution effect; P, significance value; %Var_P_ & %Var_G_, proportion of the phenotypic and genetic variances explained by the SNP.

^*^ SNPs used to create haplotypes to select parents for the assortative mating in the QTL validation experiment.

^†^ Informative SNPs in offspring from assortative mating experiment

Additionally, cross validation was performed using model 1 fitting the top four significant markers together with random polygenic effect to obtain predicted phenotypes and correlations between predicted phenotypes and observed phenotypes. The Pearson correlation value was 0.62 meaning that the four markers could predict the survival of fish with a medium reliability.

### 3.3 Challenge test of fish produced by MAS assortative mating using haplotypes

**FIGURE 3:**
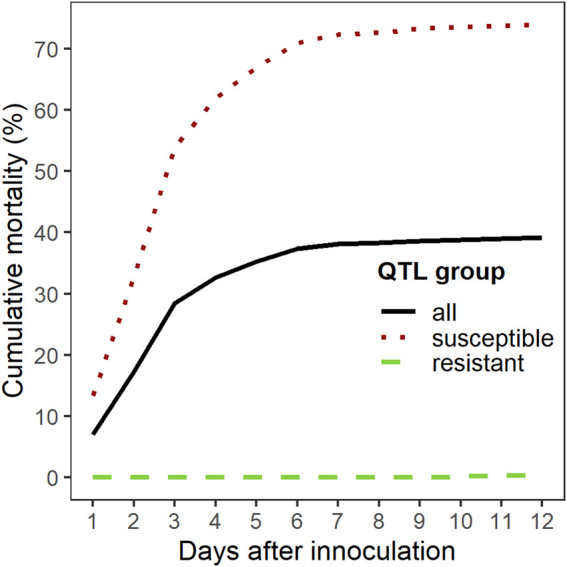
Cumulative mortality (%) for *S. iniae* in QTL validation experiment.

**FIGURE 4:**
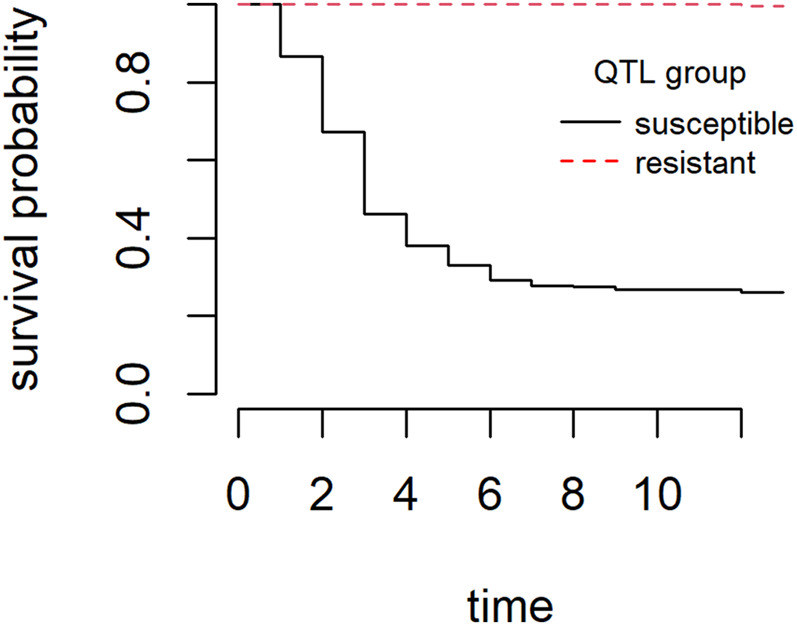
Kaplan-Meier survival curves for “resistant” group and “susceptible” group during the *S. iniae* QTL validation experiment.

The overall cumulative percent mortality at the end of the *S. iniae* challenge was 39% (Figure 3). Mortality began the day after challenge, peaked on day 3, and most mortality occurred from days 1–4 (Figure 3). Analysis of the mortality data of the two groups yielded statistically significant (Chi-square test, *p* < 0.001) differences with a final CPM of 73% for the “susceptible” group and less than 1% for the “resistant” group. Survival curves (time to death) between the two groups were also significantly different (Log-Rank tests *p* < 0.001) (Figure 4). *S. iniae* was reisolated from 100% (44/44) of the sampled fish brain tissue.

**FIGURE 5:**
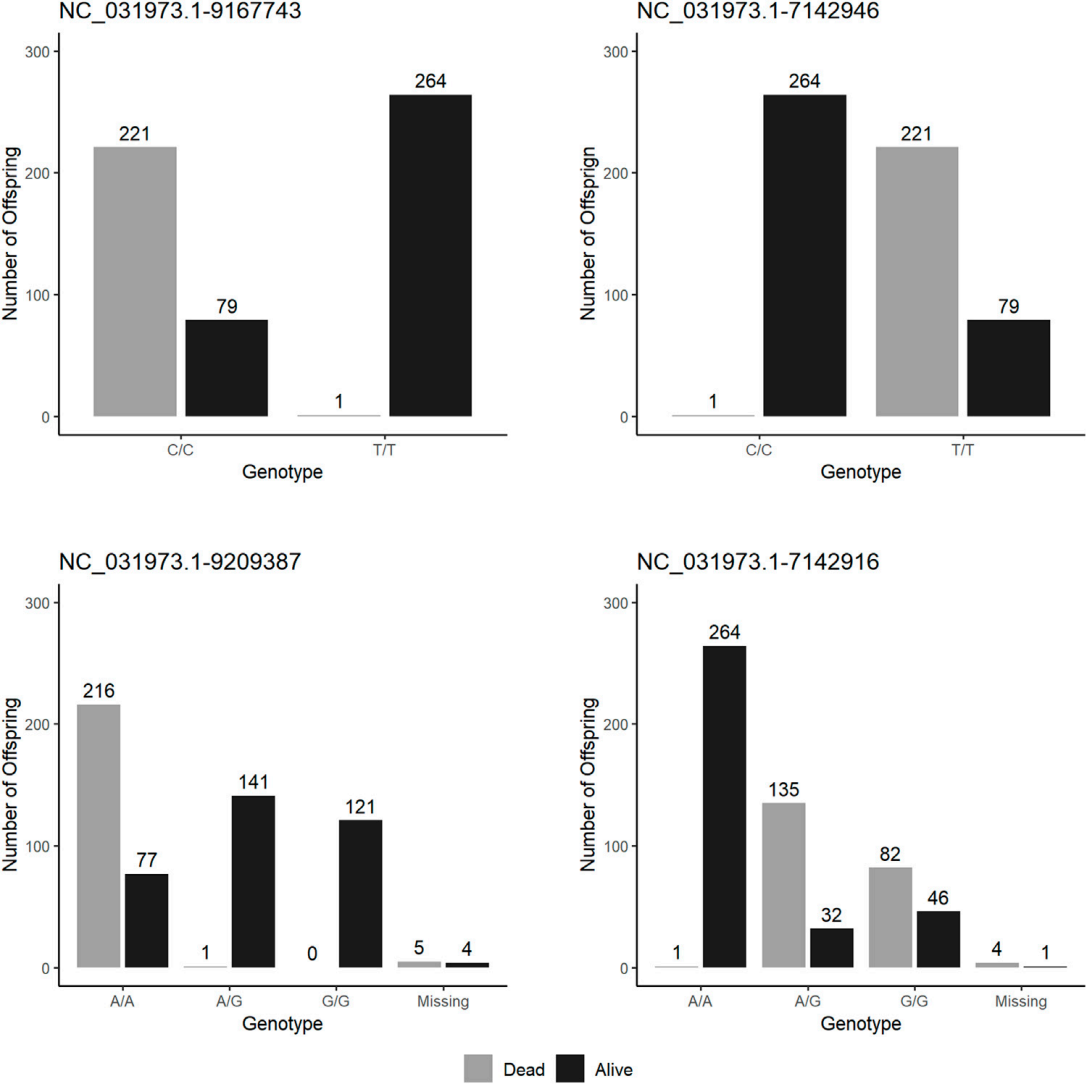
Survival to *S. iniae* at the end of test by haplotypes on the four top markers in QTL validation experiment.

After genotyping, parental assignment showed fish from “susceptible” group came from a single paternal half-sib family (i.e., one male mated to two females), and fish from the “resistant” group came from a single mating (Table 4). After QC assessment, the most significant marker for *S. iniae* resistance (NC_031973.1_9485417) was removed as it did not show polymorphism. Figure 5 shows haplotype survival of the four most significant markers which were used to select fish. Haplotypes results confirm the markers selected can predict survival for *S. iniae* accurately.

**TABLE 4:** Survival results of assortative mating for *S. iniae* QTL validation experiment (after parental assignment).

Group	Sire	Dam	Dead (N)	Alive (N)
“Resistant”	G7B1-MalR1	G7B1-FemR1	1	253
	Not Assigned	G7B1-FemR1	0	11
“Susceptible”	G7B1-MalS1	G7B1-FemS1	135	63
	G7B1-MalS1	G7B1-FemS2	86	16

Group, group into which breeders were classified based on the four selected markers; Sire, assigned sire; Dam, assigned dam; Dead & Alive, number of fish dead and alive at the end of the challenge test.

## 4 Discussion

Results from GWAS analysis for *S. iniae* suggest the presence of a novel QTL area on linkage group 8 (LG8) explaining from 12% to 26% of the genetic variance. Estimates of percentage of genetic variance explained by the SNPs may be underestimated because of the dichotomous nature of the trait (Stringer et al., 2011) and sample size. Nonetheless, the use of four top SNP markers allowed prediction of the phenotype of individuals with an accuracy of approximately 60%, which we consider enough to justify implementation of MAS in the Spring Genetics breeding program. The assortative mating experiment confirmed the feasibility of MAS, since mortality was less than 1% in offspring of fish homozygous for resistant alleles and 73% of the offspring of parents whose haplotype was characterized with susceptible alleles died during challenge test. However, the most significant allele failed to pass the genotyping QC in the offspring; thus, it was not possible to relate survival to haplotypes of the offspring. Nonetheless, when exploring haplotypes for other markers (Figure 5) it was possible to observe that the use of the fifth most significant marker would accurately select parents with QTL favourable for survival (Figure 5).

Within the entire 3.98 Mb region comprising the positions of first and last significant SNPs, genes underlying the QTL were searched using the Nile tilapia genome (Conte et al., 2017). Sixty-two genes were identified with functions mainly related to catalytic, binding, transmembrane transport and signalling receptor activity (Supplemental Table S1). Among the genes identified within the significant region on LG8, some genes were considered as candidate genes behind the QTL, either because their participation in host-pathogen interaction or importance in neuronal activity given the tropism of *S. iniae* for the central nervous system. The gene *ccr10* (chemokin receptor 10) is located about 100 kb downstream the first significant SNP, which has known chemotactic activity, and changes in expression of genes from the same family have been reported as part of the acute response of infection of *S. iniae* in Nile tilapia (Zhu et al., 2015). Gene *exoc7* (exocyst complex component 7) was located about 20 kb downstream the ninth most significant SNP; exocyst complex is known to regulate entry of bacterium into host cells (Heider and Munson, 2012). Also, the seventh most significant SNP was placed in an intronic region of gene *rnf213*, which contains a RING finger domain; proteins with a RING finger domain are involved in different cellular functions including apoptosis and has antibacterial activity (Thery et al., 2021). Likewise, three of the significant SNPs were found in intronic regions within gene *grid1b* (glutamate receptor, ionotropic, delta 1b), including the third most significant SNP. Gene *grid1b* is known to play an important role in excitatory synaptic transmission in the central nervous system (Nenadic et al., 2012). Gene *ebf3a* is located downstream of the most significant SNP in humans, homologous genes have been found to be implicated in the function of mature B cells (Vilagos et al., 2012), B cells are central the fish’s adaptative immune response (Monir et al., 2022).

Here we report the first major QTL for resistance to a bacterial disease in Nile tilapia. For salmon, significant QTLs are widely used, for example, for selection to IPN resistance (Houston et al., 2008; Moen et al., 2009; Moen et al., 2015) and pancreas disease (PD) (Gonen et al., 2015). Also, a QTL explaining 6% of the variation was found for resistance to infectious salmon anemia virus (ISA) (Moen et al., 2007) in Atlantic salmon and more recently for resistance for *Piscine myocarditis virus* (CMS) (Boison et al., 2019; Hillestad et al., 2020) which are also used for MAS.

No significant correlation has been found between survival to *S. iniae* and harvest weight (LaFrentz et al., 2020), meaning selection for survival to *S. iniae* will likely not impact selection for harvest weight, probably the main trait under selection in Nile tilapia breeding programs.

## 5 Conclusion

This study confirms the presence of a QTL with a considerable effect on survival to *S. iniae* in Nile tilapia and demonstrates that MAS is an appropriate tool for improving resistance to *S. iniae* in this population to reduce incidence of this disease in the farms.

## Data Availability

The raw genotype data are not publicly available as it is the property of a commercial enterprise. Requests to access the raw genotype material should be directed to SV-A at sergio.vela@bmkgenetics.com.

## Footnotes

1 bcl2fastq Conversion Software https://support.illumina.com/sequencing/sequencing_software/bcl2fastq-conversion-software.html

2 Picard software https://broadinstitute.github.io/picard/
