# Supplementary material for: Genome-wide association study for Streptococcus iniae in Nile tilapia (Oreochromis niloticus) identifies a significant QTL for disease resistance
Source: Front Genet. 2023 Mar 2;14:1078381. doi: 10.3389/fgene.2023.1078381 (PMC10017449; doi:10.3389/fgene.2023.1078381)
Supplement: Supplementary file 1 [file DataSheet1.PDF]

Suplementary file

Table S.1 Genes that were identified within the significant region of linkage group 8 (LG8).

Gene ID	Symbol	Gene name
100695225	grb7	growth factor receptor bound protein 7
100694955	LOC100694955	SRC kinase signaling inhibitor 1
102080818	LOC102080818	keratin, type I cytoskeletal 9-like
100694697	fmnl1b	formin-like 1b
100694427	ccr10	chemokine (C-C motif) receptor 10
106098479	LOC106098479	UDP-glucuronosyltransferase 2B31
109203178	LOC109203178	UDP-glucuronosyltransferase 2B31 pseudogene
100699395	LOC100699395	endonuclease domain-containing 1 protein
100694160	LOC100694160	DELTA-stichotoxin-Hcr4a-like
100693886	LOC100693886	bryoporin-like
100693623	plaub	plasminogen activator, urokinase b
100699128	hid1b	HID1 domain containing b
102081765	LOC102081765	proton channel OTOP3
100698861	LOC100698861	Usher syndrome type-1G protein homolog
102082090	fads6	fatty acid desaturase 6
100693358	trim16	tripartite motif containing 16
100698590	tvp23b	trans-golgi network vesicle protein 23 homolog B
100697612	exoc7	exocyst complex component 7
100693087	LOC100693087	galanin receptor type 2
100692811	kcnj16	potassium inwardly rectifying channel subfamily J member 16
100692542	kcnj2a	potassium inwardly rectifying channel subfamily J member 2a
100697345	map2k6	mitogen-activated protein kinase kinase 6
100692271	LOC100692271	BTB/POZ domain-containing protein 17
100692002	gpr142	G protein-coupled receptor 142
100691732	gprc5c	G protein-coupled receptor, class C, group 5, member C
100697073	oxld1	oxidoreductase-like domain containing 1
100696806	pde6gb	phosphodiesterase 6G, cGMP-specific, rod, gamma, paralog b
100691464	LOC100691464	gastrula zinc finger protein XlCGF57.1
100691195	LOC100691195	zinc finger and SCAN domain-containing protein 12
102078572	LOC102078572	gastrula zinc finger protein XlCGF57.1
102077854	LOC102077854	zinc finger protein OZF
100690930	LOC100690930	oocyte zinc finger protein XlCOF6.1
100696539	nploc4	NPL4 homolog, ubiquitin recognition factor
102077096	rnf213b	ring finger protein 213b
100690659	cep131	centrosomal protein 131
100690385	st6galnac	ST6 (alpha-N-acetyl-neuraminyl-2,3-beta-galactosyl-1,3)-N-acetylgalactosaminide alpha-2,6-sialyltransferase
102076345	LOC102076345	zinc finger protein OZF
109203183	LOC109203183	serine/threonine-protein kinase pim-1

106098477	LOC106098477	gastrula zinc finger protein XlCGF57.1
102076612	LOC102076612	zinc finger protein OZF
100712205	aatkb	apoptosis-associated tyrosine kinase b
100711936	baiap2b	BAR/IMD domain containing adaptor protein 2b
100705156	chmp6b	charged multivesicular body protein 6b
109203184	LOC109203184	zinc finger protein 37
102083040	LOC102083040	cilia- and flagella-associated protein 251
100711392	LOC100711392	zinc finger protein 2 homolog
109203181	LOC109203181	gastrula zinc finger protein XlCGF57.1
100710590	LOC100710590	gastrula zinc finger protein xFG20-1
100710859	LOC100710859	gastrula zinc finger protein XlCGF57.1
102081504	LOC102081504	zinc finger protein 665
102081668	LOC102081668	endothelial zinc finger protein induced by tumor necrosis factor alpha
100705950	abca5	ATP-binding cassette, sub-family A (ABC1), member 5
100711123	asb3	ankyrin repeat and SOCS box containing 3
100711393	lg8h10orf88	linkage group 8 C10orf88 homolog
100711666	htra1b	HtrA serine peptidase 1b
100711938	plekha1b	pleckstrin homology domain containing, family A (phosphoinositide binding specific) member 1b
100712206	LOC100712206	tripartite motif-containing protein 35
100712474	LOC100712474	wings apart-like protein homolog
100689855	atoh1c	atonal bHLH transcription factor 1c
100690120	grid1b	glutamate receptor, ionotropic, delta 1b
100692543	LOC100692543	microfibril-associated glycoprotein 4-like
